# Metagenomic next-generation sequencing versus traditional laboratory methods for the diagnosis of central nervous system opportunistic infections in HIV-infected Chinese adults

**DOI:** 10.1038/s41598-023-31974-1

**Published:** 2023-03-25

**Authors:** Liping Deng, Qian Li, Wenjia Hu, Xiaoping Chen, Tielong Chen, Shihui Song, Pingzheng Mo, Shi Zou, Yongxi Zhang, Shicheng Gao, Liangjun Chen, Ke Zhuang, Rongrong Yang, Yong Xiong

**Affiliations:** 1 Department of Infectious Diseases, Zhongnan Hospital of Wuhan University, Wuhan, Hubei China; 2 Department of Laboratory Medicine, Zhongnan Hospital, Wuhan University, Wuhan, Hubei China; 3 ABSL‐III Laboratory at the Center for Animal Experiment, State Key Laboratory of Virology, Wuhan University, Wuhan, Hubei China

**Keywords:** Diseases, Medical research

## Abstract

To evaluate clinical value of metagenomic next-generation sequencing (mNGS) in people living with HIV/AIDS (PLWHA) who had CNS disorders. Cerebrospinal fluid (CSF) samples from 48 PLWHA presenting with CNS disorders were sequenced using mNGS and compared with clinical conventional diagnostic methods. In total, 36/48 ss(75%) patients were diagnosed with pathogen(s) infection by mNGS, and the positive detection proportion by mNGS was higher than that by clinical conventional diagnostic methods (75% vs 52.1%, *X*^*2*^ = 5.441, *P* = 0.020). Thirteen out of 48 patients (27.1%) were detected with 3–7 pathogens by mNGS. Moreover, 77 pathogen strains were detected, of which 94.8% (73/77) by mNGS and 37.0% (30/77) by clinical conventional methods (*X*^*2*^ = 54.206, *P* < 0.001). The sensitivity and specificity of pathogens detection by mNGS were 63.9% (23/36) and 66.7% (8/12), respectively, which were superior to that by clinical conventional methods (23/36 vs 9/25, *X*^2^ = 4.601, *P* = 0.032; 8/12 vs 5/23, *X*^2^ = 5.029, *P* = 0.009). The application of mNGS was superior for its ability to detect a variety of unknown pathogens and multiple pathogens infection, and relatively higher sensitivity and specificity in diagnosis of CNS disorders in PLWHA.

## Introduction

HIV infection can gradually lead to immune deficiency and lead to a variety of opportunistic infections^1^. Central nervous system (CNS) infectious remain a major cause of morbidity and mortality worldwide among people living with HIV/AIDS (PLWHA), particularly in those with advanced immunosuppression^2–5^. Many PLWHA are unaware of their HIV infection status, resulting in delayed clinical diagnosis and treatment^3^. About 10–20% PLWHA have neurologic symptoms as an initial manifestation and the reason for hospitalization^6^, which contribute to high morbidity and mortality in combination antiretroviral therapy (cART)-naive individuals in developing countries^7,8^. Except for immune deficiency status, physical and chemical mutagens have been reported to be associated with multiple neurological disorders in humans^9,10^. Therefore, early identification of pathogens or exclusion of CNS infection is of great importance to reduce morbidity and mortality, and ultimately prolong the life expectancy or improve quality of life.

For patients with suspected diagnosis of CNS infection, lumbar puncture (LP) is a routine operation and cerebrospinal fluid (CSF) is commonly collected for clinical conventional testing, which are generally limited to CSF routine and biochemical tests, smear, culture and molecular assays^11^. Although these clinical conventional testing for one or a few common pathogens are available, a timely and precise diagnosis of the aetiology of CNS infection remains challenging, especially for those with multiple or rare pathogen infections.

Metagenomic next-generation sequencing (mNGS) is a new and promising approach to identify co-infections in an unbiased manner by a single assay. The use of mNGS can overcome the limitations of current diagnostic tests, allowing for hypothesis-free, culture-independent, pathogen detection directly from clinical specimens regardless of the type of microbe; mNGS can even be used for novel organism discovery. Currently, mNGS has been applied in many infectious diseases due to its superiority in unconventional pathogens, novel pathogens, and mixed infections^12–15^. Immunodeficiency is the most prominent characteristic of PLWHA, which leads to atypical neurologic symptoms or radiographic patterns. CNS infections in PLWHA are often challenging to diagnose by traditional microbiological testing, impacting treatment and outcome. Therefore, mNGS may be especially advantageous in the diagnosis of CNS diseases in PLWHA. However, due to its relatively high economical burden, the performance of mNGS for the diagnosis of CNS infection among PLWHA in resource-limited areas is still not well evaluated. Consequently, this study aimed to evaluate the diagnostic performance of mNGS in PLWHA with CNS diseases. Cerebrospinal fluid (CSF) samples from 48 PLWHA presenting with CNS disorders were sequenced using mNGS and compared with clinical conventional diagnostic methods.

## Methods

### Population and Study Design

This was a hospital-based study, and it was conducted at Zhongnan Hospital of Wuhan University, China, between January 2021 and January 2022. As described in a previous study^16^, PLWHA were included if they (1) had three or more symptoms of meningitis and/or encephalitis, including headache, seizure, nausea/vomiting, photophobia, alteration in consciousness or a focal visual field defect; and (2) agreed to do a lumbar puncture(LP) to collect CSF for mNGS and clinical conventional testing. Exclusion criteria for enrollment included: (1) patients with delirium, such as sepsis due to a non-neurological infection or metabolic abnormality; (2) patients with peripheral neuropathy and psychosis rather than medical explanation for their symptoms; (3) patients with SARS-CoV-2 infection; or (4) patients with a documented immunosuppressive except for HIV infection or neurosurgical illness.

All patients who were suspected to have CNS infection in this study received lumbar puncture for CSF routine test and biochemical tests. As described previously in a Chinese study^17^, CSF conventional testing included bacterial and fungal smear; acid-fast stain; and culture of bacteria, fungal organisms and Mycobacterium species. As cryptococcal meningitis and tuberculous meningitis or encephalitis are common in PLWHA^18^, cryptococcal antigen test, Chinese ink staining and GeneXpert MTB/RIF tests were requested. Except for blood and CSF assays, CT or MRI scan of brain was also part of the routine procedures. Meanwhile, another CSF sample was collected for mNGS. The detected pathogens through clinical conventional testing available in this hospital would be further compared with the results by mNGS.

Two physicians were responsible for checking and analyzing clinical information, including age, gender, marital status, risk factors for HIV infection, etc. This study was approved by the Ethics Committee of Zhongnan Hospital of Wuhan University (Research Ethics No. 2021079). The study protocol was conducted in accordance with the latest version of the Declaration of Helsinki. The written informed consent was signed from all participants included in this study.

### Library preparation and metagenomic sequencing

DNA library was prepared by automatic nucleic acid extraction, enzymatic fragmentation, end repair, terminal adenylation and adaptor ligation according to a previous study^19^. Finished libraries were quantified by real-time PCR (KAPA) and pooled. Shotgun sequencing was carried out on illumina Nextseq. Approximately 20 million of 50 bp single-end reads were generated for each library. Bioinformatic analysis was conducted as described in a previous report^20^. Briefly, sequences of human origin were filtered (GRCh38.p13) and the remaining reads were aligned to a reference database (NCBI nt, GenBank and in-house curated genomic database) to identify the microbial species and read count. For each sequencing run, a negative control (culture medium containing 104 Jurkat cells/mL) was included. DNA extraction and library preparation were conducted on the NGS Automatic Library Preparation System (Cat. MAR002, MatriDx Biotech Corp. Hangzhou, China). Reagents included: Nucleic Acid Extraction Kit (Cat. MD014, MatriDx Biotech Corp. Hangzhou, China), Total DNA Library Preparation Kit (other samples).

### mNGS reporting criteria

Microbial reads identified from a library were reported if: (1) the sequencing data passed quality control filters (library concentration > 50 pM, Q20 > 85%, Q30 > 80%); (2) negative control (NC) in the same sequencing run does not contain the species or the RPM (sample)/RPM (NC) ≥ 5, which was determined empirically according to previous studies^19,21,22^ as a cutoff for discriminating true-positives from background contamination. After excluding common skin colonized bacteria, reagents or environmental background contamination and index hopping, pathogen-positive microbiome or suspected contaminated colonizing bacteria was distinguished.

### Definition of specificity and sensitivity

Based on clinical presentations and accessible results during hospitalization, a tentative diagnosis was made for subsequent empirical treatment. After a course of standardized treatment, further estimate was made according to the effectiveness of the treatment, and final diagnosis was determined at discharge. To guaranteed the accuracy and reliability of discharge diagnosis, trained infectious physician, radiologists and neurologists were all invited to take part in the group discussion. Both the results of mNGS and clinical conventional methods were compared with discharge diagnosis to assess the sensitivity and specificity of each detection method.

### Statistical analysis

All statistical analyses were performed using SPSS Statistics version 23.0 software. Categorical variables were described as frequency rates and percentages, and quantitative data were expressed as mean ± SD. Comparisons between groups were analyzed by chi-square test or Fisher’s exact test for categorical variables and by Student’s *t*-test for quantitative variables. *P* < 0.05 was considered statistically significant.

### Ethical approval and consent to participate

This study was approved by the Ethics Committee of Zhongnan Hospital of Wuhan University (Research Ethics No. 2021079). The written informed consent was signed from all participants included in this study.

## Results

### Demographics and clinical characteristics

**Figure 1:**
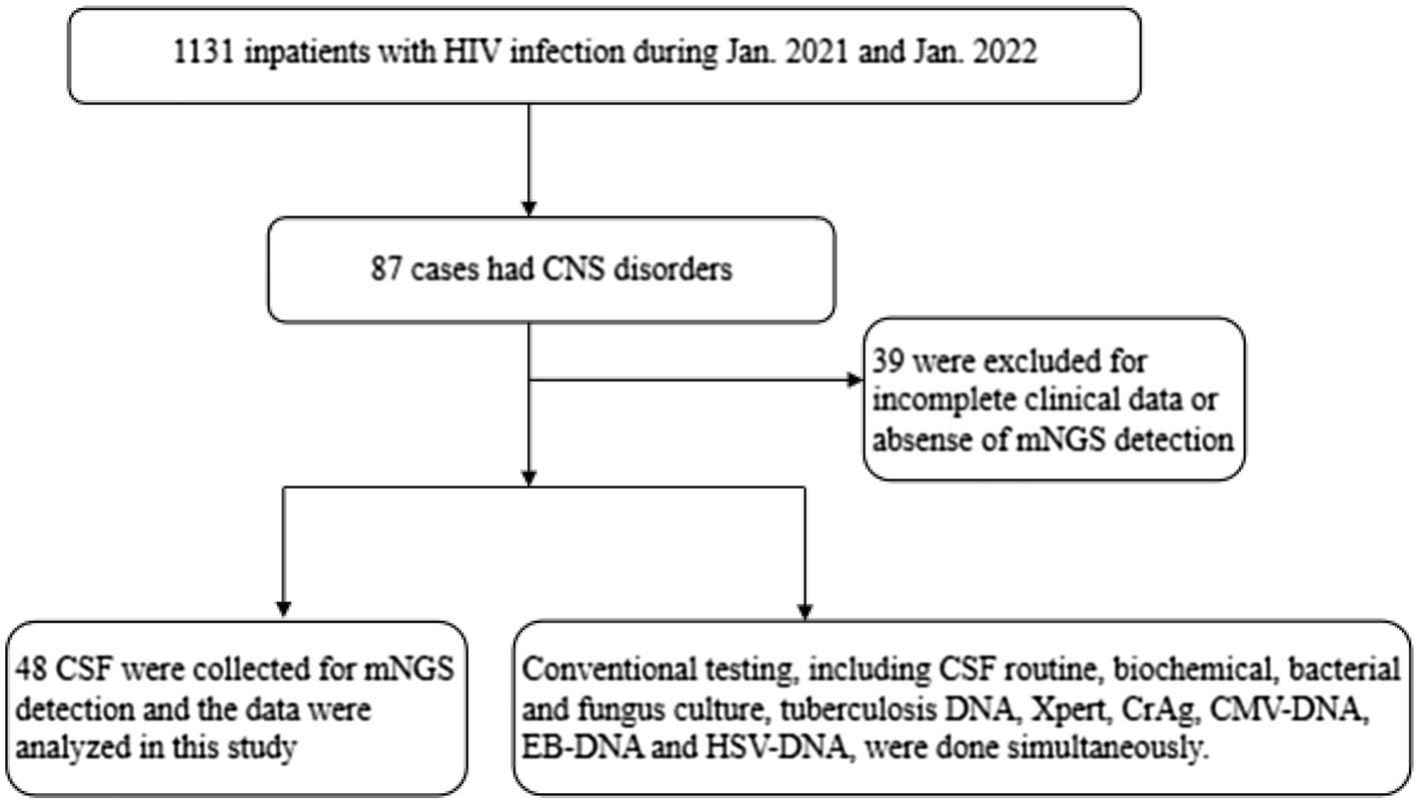
Study profile.

Among 1131 PLWHA who were hospitalized in Zhongnan Hospital of Wuhan University from Jan 1, 2021 to Jan 1, 2022, 48 patients with a complaint of new or recurrent neurological or psychiatric symptoms were included in this study (Fig. 1). Overall, the majority of individuals were male, 91.7% (44/48) acquired HIV infection by sexual contact, 62.5% (30/48) had underlying diseases, and 81.3% (39/48) were receiving cART(Table 1).

**Table 1:** Characteristics of 48 inpatients who had done mNGS testing.

	No. (%)	Mean ± SD/Median (IQR)
Age		40.3 ± 13.4
Male	44 (91.7)	
Marital status		
Married	25 (52.1)	
Single	18 (37.5)	
Divorced/Widowed	5 (10.4)	
Past medical history		
HBV infection	6 (12.5)	
HCV infection	3 (6.3)	
Syphilis	16 (33.3)	
Tuberculosis	3 (6.3)	
Cardiovascular and cerebrovascular diseases	2 (4.2)	
Routes of HIV acquisition		
Sexually transmission	44 (91.7)	
Blood-borne transmission	4 (8.3)	
cART coverage	39 (81.3)	
Symptoms and signs		
Headache	24 (50.0)	
Fever	23 (47.9)	
Nausea	15 (31.3)	
Consciousness disorder	8 (16.7)	
Blurred vision	6 (12.5)	
Seizure	5 (10.4)	
Dyspnea	1 (2.1)	
Neck stiffness	1 (2.1)	
Days from illness onset to first admission		20 (14, 30)
Days of hospital stay		17 (14, 25)
LgHIV-VL		4.4 (3.6, 5.1)
Died within 12 weeks	4 (8.3)	
Abnormal CT or MRI scan of brain	38 (79.2)	
Blood routine and biochemistry results		
White blood cell count, × 10^9^/L		4.22 ± 1.96
< 4.0 × 10^9^/L	22 (45.8)	
> 10.0 × 10^9^/L	1 (2.1)	
Neutrophil count, × 10^9^/L		2.7 (1.4, 3.8)
Lymphocyte count, × 10^9^/L		0.7 (0.4, 1.2)
< 1.0 × 10^9^/L, No. (%)	26 (54.2)	
Hg, g/L		109.8 ± 25.4
< 90 g/L	9 (18.8)	
Platelet count, × 10^9^/L		181.9 ± 96.2
< 100 × 10^9^/L	11 (23.0)	
Alanine aminotransferase, U/L		21 (14.3, 39.3)
> 50U/L	9 (18.8)	
Aspartate aminotransferase, U/L		27 (19.3, 49.8)
> 40U/L	14 (29.2)	
Albumin, g/L		33.7 (27.2, 9.6)
< 40 g/L	39 (81.3)	
Globulin, g/L		35.8 ± 6.5
> 30 g/L	40 (83.3)	
Creatinine, μmol/L		5.6 (4.1, 7.4)
> 104 μmol/L	6 (12.5)	
Urea nitrogen, mmol/L		5.6 (4.1, 7.4)
> 7.6 mmol/L	10 (20.8)	
CYSC, mg/L		1.0 (0.9, 1.2)
> 1.2 mg/L	14 (29.2)	
Lymphocyte subsets and NK cells in peripheral blood		
CD3 + T cell		370.5 (242.0, 690.5)
CD3 + CD4 + %Lym		6.9 (3.8, 10.0)
CD3 + CD4 + Abs		34.0 (13.0, 122.5)
CD3 + CD8 + %Lym		61.2 (53.3, 61.2)
CD3 + CD8 + Abs		406.5 (208.5, 564.0)
CD4 + /CD8 + Ratio		0.12 (0.07, 0.18)
CD19 + %Lym		9.0 (5.0, 14.3)
CD19 + B cell		58.5 (22.5, 77.5)
CD16 + CD56 + % NK cell		11.0 (7.3, 16.3)
CD16 + CD56 + Abs		76.5 (39.0, 104.0)
Lactate dehydrogenase, U/L		211.0 (169.0, 480.5)
> 243U/L	18 (37.5)	
D-dimer, ng/mL		440.0 (205.0, 854.8)
> 500 ng/mL	22 (45.8)	
Infection-related biomarkers		
ESR, mm/h		34.9 ± 23.9
> 15 mm/h	31 (64.6)	
C-reactive protein, mg/L		20.0 (5.8, 71.6)
> 10 mg/L	17 (35.4)	
Interleukin-6, pg/mL		11.6 (6.4, 31.6)
> 7 pg/mL	25 (52.1)	
Procalcitonin, ng/mL		0.06 (0.05, 0.32)
> 1.0 ng/mL	7(14.6)	

The top five neurological symptoms were headache (50.0%), fever (47.9%), nausea (31.3%), consciousness disorder (16.7%) and blurred vision (12.5%). Average time from onset of symptoms to first admission and hospital stay were 20 days and 17 days, respectively. The median CD4^+^ T lymphocyte counts and Log_10_HIV-RNA(IQR) were 34/ul and 4.4 (3.6, 5.1), respectively. The proportion of individuals who had abnormal CT or MRI scan of brain was 79.2% (38/48). Four patients were died during hospitalization or within 12 weeks of follow-up. The results of blood routine and biochemistry, infection-related biomarkers, lactate dehydrogenase and D-dimer were shown in Table 1.

### Number of pathogens detected in CSF by mNGS

**Figure 2:**
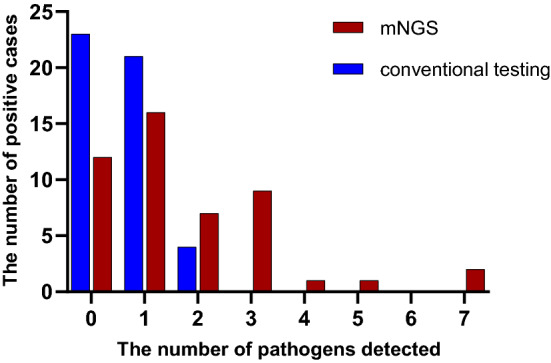
The distribution of pathogens number detected by two methods.

In this study, 75% (36/48) of the pathogens were detected by mNGS, whereas 52.1% (25/48) by clinical conventional method, and the difference between the two methods was statistically significant (*X*^*2*^ = 5.441, *P* = 0.020). Moreover, the detection rates of two or more pathogens by mNGS was 41.7% (20/48), which was much higher than 8.3%(4/48) by clinical conventional method (*X*^*2*^ = 14.222, *P* < 0.001). It's worth noting that 13 out of 48 patients (27.1%) were detected with 3–7 pathogens infection by mNGS, whereas none by clinical conventional methods. The distribution of the number of pathogens detected by mNGS and clinical conventional method was shown in Fig. 2.

### Types of pathogens detected in CSF by mNGS

The top 3 pathogens detected by mNGS were Epstein-Barr virus(EBV), CMV and Cryptococcus neoforman, which were consistent with the distribution of common pathogens detected by clinical conventional methods. The detection efficiency of the above three common pathogens by mNGS was 72.9% (35/48), which was slightly superior than that by clinical conventional methods (72.9% vs 56.3%, *X*^2^ = 2.915, *P* = 0.088). In this study, the significant superiority of mNGS was that 26 species with a total of 40 pathogens were detected by mNGS but not detected by clinical conventional methods.

**Figure 3:**
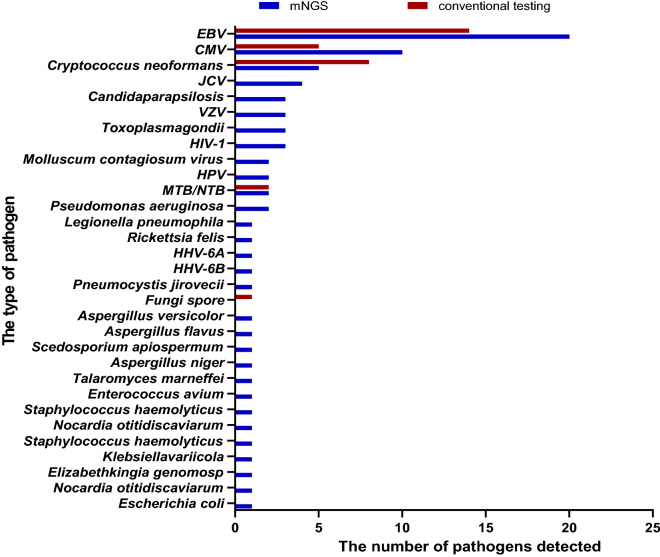
The distribution of pathogens types detected by two methods.

The results showed that the detection efficiency of cryptococcus neoformans (5/48 vs 8/48), mycobacterium tuberculosis and non-tuberculous mycobacterium (2/48 vs 2/48) by mNGS was comparable to that by clinical conventional methods. Nonetheless, we have noticed that fungal spores were detected using conventional methods rather than mNGS in 1 patient. The distribution of types of pathogens detected by mNGS and clinical conventional method was shown in Fig. 3.

### Detection efficiency of various pathogens by mNGS

Overall, 77 pathogen strains were found by combined detection of mNGS and clinical conventional methods. Among them, 73 and 30 pathogen strains were found by mNGS and clinical conventional methods, respectively. The rate of pathogens detection by mNGS (94.8%) was significantly higher than that by clinical conventional methods(39.0%) (*X*^2^ = 54.206, *P* < 0.001). Twenty-six pathogen strains were found by both methods, which accounted for 33.8% (26/77). Among 73 pathogen strains detected by mNGS, 47 were missed diagnosis by clinical conventional methods. The false negatives detection rate by clinical conventional methods was 64.4% (47/73). Meanwhile, among 30 pathogen strains detected by clinical conventional methods, 4 were missed diagnosis by mNGS. Overall, the false negatives detection rate by mNGS was 13.3% (4/30), which was significantly lower than that by clinical conventional methods (*X*^2^ = 22.167, *P* < 0.001).

Except for fungi, the number of patients detected with virus, bacteria and atypical pathogens infection by mNGS was more than that by clinical conventional methods. Moreover, compared with clinical conventional methods, more pathogen strains were detected by mNGS. These data were shown in Table 2.

**Table 2:** Detection efficiency comparison of various pathogens by mNGS and clinical conventional testing.

	mNGS	Clinical conventional testing	*X*^2^	*P*
Virus				
Detected cases of infection (n,%)	32 (66.7)	17 (35.4)	9.379	0.002
Positive detection rate of virus strains	42/46 (91.3)	19/46 (41.3)	25.737	0.000
Detection rate of virus strains not detected by control group	23/42 (54.8)	0/19 (0)	16.702	0.000
Bacteria				
Detected cases of infection (n,%)	8 (16.7)	0	6.682	0.010
Positive detection rate of bacteria strains	16/16 (100.0)	2/16 (12.5)	24.889	0.000
Detection rate of bacteria strains not detected by control group	14/16 (87.5)	0/2 (0)	7.875	0.005
Fungi				
Detected cases of infection (n,%)	10 (20.8)	8 (16.7)	0.274	0.601
Positive detection rate of fungi strains	10/14 (71.4)	9/14 (64.3)	0.164	0.686
Detection rate of fungi strains strains not detected by control group	5/10 (50.0)	4/9 (44.4)	0.059	0.809
Atypical pathogen				
Detected cases of infection (n,%)	5 (10.4)	0	4.444	0.035
Positive detection rate of atypical pathogen strains	5/5 (100.0)	0/5 (0)	10.000	0.002
Detection rate of atypical pathogen strains not detected by control group	5/5 (100.0)	0 (0)	–	–
Total detection rate of pathogen strains not detected by control group	47/73 (64.4)	4/30 (13.3)	22.167	0.000

### Significance of mNGS results for clinical diagnosis and treatment

According to the results by mNGS and clinical conventional methods, clinical diagnosis was made and targeted treatment measures were given. After follow-up and clinical efficacy verification, the sensitivity and specificity of pathogens detection by mNGS were 63.9% (23/36) and 66.7% (8/12), respectively, which were superior to that by clinical conventional methods (23/36 vs 9/25, *X*^2^ = 4.601, *P* = 0.032; 8/12 vs 5/23, *X*^2^ = 5.029, *P* = 0.009).

**Figure 4:**
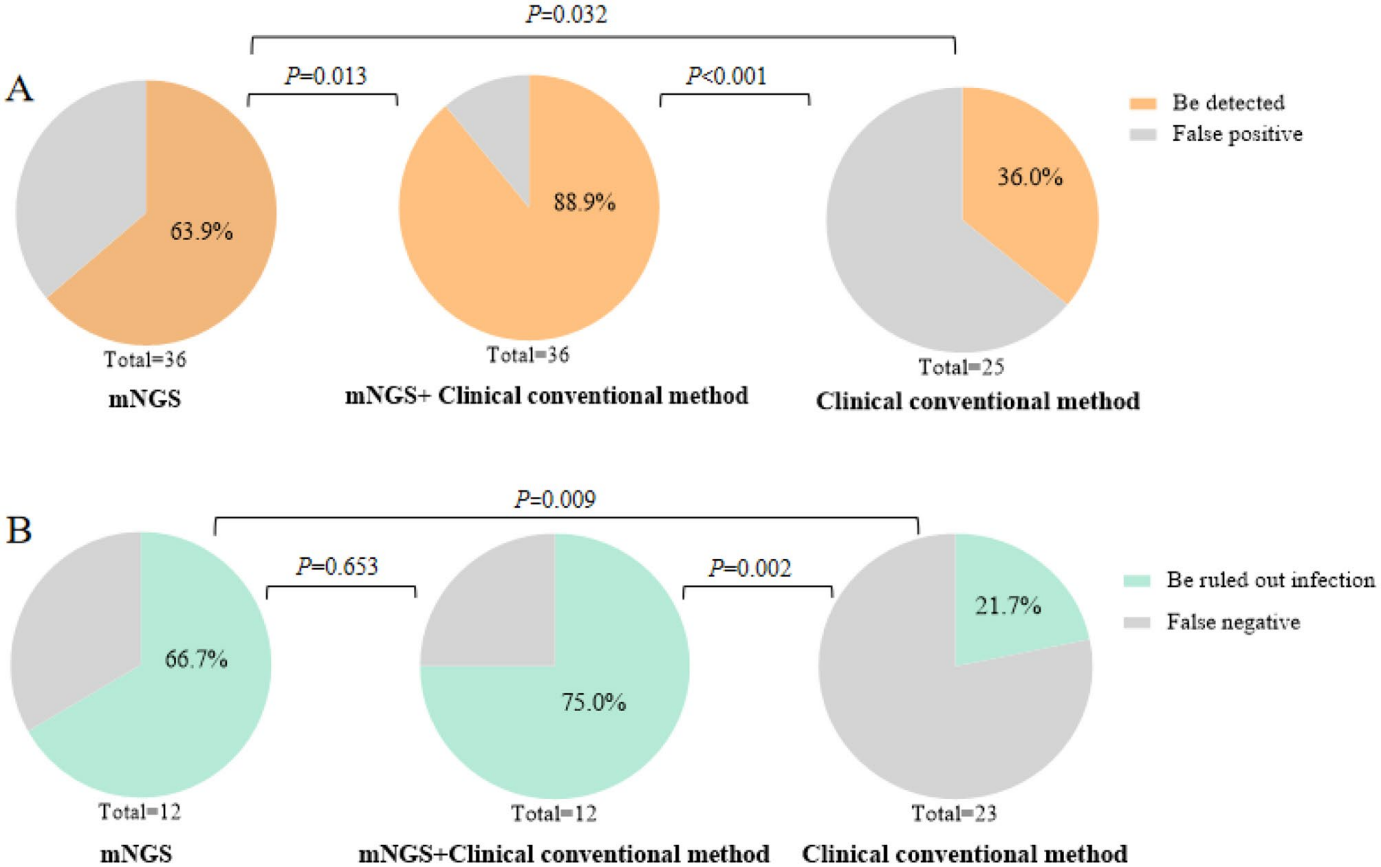
The sensitivity and specificity of mNGS and clinical conventional testing in diagnosis of CNS disorders in PLWHA. (**A**) Comparison of sensitivity; (**B**) Comparison of specificity.

Through combination analysis of the results by mNGS and clinical conventional methods, the effective rate of positive results to guide clinical diagnosis and treatment was 88.9%, while the effective rate of negative results to rule out pathogens infection was 75%. Both positive and negative results based on comprehensive analysis of the two methods were more effective for clinical diagnosis and treatment than either single method (Fig. 4).

### Analysis of death causes

During hospitalization or within 12 weeks of follow-up, 4 out of 48 patients died, and the mortality was 8.3%. All the four patients had a definitive diagnosis but died due to serious illness that could not be effectively controlled. Not by clinical conventional methods but by mNGS, one patient was diagnosed to be infected with a variety of pathogens, including Marneffei and Epstein-Barr virus. The other three patients were confirmed with the same pathogens infection both by mNGS and clinical conventional methods. The diagnostic methods and specific causes of death were shown in Table 3.

**Table 3:** Analysis of causes of death.

	Diagnostic methods		Death causes	Other co-infections
	mNGS	Clinical conventional methods		
Patient 1	√	√	Burkitt's lymphoma	No
Patient 2	√	√	Cryptococcal meningitis	No
Patient 3	√	√	Cryptococcal meningitis	No
Patient 4	√	×	Penicillium marneffei disease	EB virus

## Discussion

mNGS is a new technology that make it possible to detect and identify an unlimited range of organisms in a single testing. Furthermore, it is important that mNGS includes genetic information on detected pathogens. Due to the independence of organism cultivation, the compatibility of genomic diversity or mutations, and the universality of pathogens that can be detected, mNGS offers several superiority that conventional tests could not achieve. To our knowledge, in resource-limited areas, studies about clinical application of mNGS in identifying pathogens of CNS infection in PLWHA is very limit. This real-world study might provide data and help clinicians to comprehensively and objectively grasp the clinical value of mNGS in PLWHA with CNS infections.

In this study, the pathogen detection rate by mNGS was high and the results had a guiding role in clinical diagnosis and treatment, which proved that mNGS remained an effective detection means for suspected CNS infection even among immunodeficient population. The results were consistent with previous studies^13,17^. Undoubtedly, mNGS was more advantageous in guiding clinical diagnosis and treatment when clinical manifestations of opportunistic infections were atypical due to immune deficiency by HIV infection.

The top 3 pathogens detected by mNGS were consistent with the distribution of common pathogens detected by clinical conventional methods. It was challenging to confirm the causative pathogen since similar symptoms could be present even with different pathogen infection, and nonspecific laboratory results and atypical imaging were common in PLWHA^23,24^. On the premise of proven reliability of clinical diagnosis and treatment, mNGS was more prominent in identifying unknown or unexpected pathogens, which can greatly improve the efficiency of pathogen diagnosis.

Multiple pathogens infection which ranging from 15.9% to 36.0%, was common in PLWHA^17,25^. Surprisingly, 33 out of 48 patients (68.8%) were detected with 2–7 pathogens by mNGS in this study, which was much higher than other studies^17,25^. Real-time polymerase chain reaction (PCR) based on pathogen-specific primers was used in a Zambian study^25^ and patients who were tested negative by all clinical conventional methods were included in Chen et al. study^17^. The difference of detection methods and observation subjects may account for the higher rates of multiple pathogens infection in this study.

Like all laboratory tests, the interpretation of mNGS results should be done in combination with clinical data^26^, and the accuracy of the results should ultimately depend on effect to targeted treatment based on the etiological results of mNGS. Different from previous studies, we compared and classified the detection efficiency of mNGS according to the types of pathogens. We found that mNGS was significantly superior to clinical conventional method in bacteria, viruses and atypical pathogens detection, but the detection efficiency of fungal infection was slightly lower. Physicians should make comprehensive assessment on whether or under what circumstances mNGS should be recommended in combination with the specific clinical and financial conditions of patients, and the cost-effectiveness of mNGS^27^.

Although mNGS have significant superiority in the diagnosis of viral, bacterial and atypical pathogens, its disadvantages in detecting tuberculosis or non-tuberculous mycobacterium should also be noted. The result in this study showed that the detection rate of mNGS was similar to that of clinical conventional methods. The result was a little better than that in another study, in which mycobacterium were indeed missed and still only diagnosed by clinical conventional methods^17^. From these studies, we can come to a same conclusion that, Gene Xpert and Xpert Ultra were the most appropriate choice for patients with clinically suspected tuberculosis or non-tuberculous mycobacterium infection due to its relatively higher sensitivity and more convenience^28–30^. In addition to mycobacterium, fungal espectially cryptococcus neoformans were missed detected by mNGS in this study. In HIV-negative patients, the sensitivity of mNGS were higher than those of India ink and culture, but slightly lower than those of CrAg tests^31,32^. This was consistent with the result of this study, in which some confirmed meningitis cases by clinical conventional methods were missed diagnosis by mNGS. To date, CrAg EIA was proved to be the diagnostic assay with highest sensitivity and specificity in screening cryptococcus neoformans infection in HIV-negative patients. In PLWHA likewise, the application of clinical conventional method, including CrAg EIA, together with India ink staining and culture methods, could be considered as an effective tool to clarify a diagnosis of cryptococcal meningitis.

In sub-Saharan Africa, CNS infections in PLWHA contributed to 15–25% of AIDS-related deaths^33–35^. Through the combination of mNGS and clinical conventional methods, a relatively lower risk of deaths caused by delayed targeted treatment due to missed diagnosis was found in this study. Nevertheless, early diagnosis remains the necessary and effective measure to reduce the mortality in PLWHA with CNS infections^13^.

We recognize that our study has some limitations. Due to the relatively high economical burden for mNGS and the lower acquisition of informed consent for lumbar puncture, the sample in this prospective clinical study was small, which would needs relatively longer time to complement this limitation. In addition, different from the medical reality in many western countries, the detection of JCV and VZV by PCR in CSF has not yet been incorporated into routine clinical testing in China yet, which may over-evaluated the superiority of mNGS in this real-world clinical study. This limitation can be compensated through adding these common in PLWHA but unimplemented detection items in CSF in further studies.

In conclusion, compared with clinical conventional methods, the application of mNGS in PLWHA with CNS disorders was superior for its ability to detect a variety of unknown pathogens and higher detection rate of multiple pathogens infection. The combination of mNGS and clinical conventional methods can improve the sensitivity and specificity of clinical diagnosis of CNS disorders in PLWHA. For those patients who are unable to get effective clues through conventional clinical diagnostic methods but are affordable to mNGS, the study can be duplicated in other countries or other regions with relative ease of access.

## Data Availability

The datasets used or analyzed during the current study are available from the corresponding author on reasonable request.

## References

[1] Basida SD, Basida B, Zalavadiya N, Trivedi AP (2021). Dermatological opportunistic infections in HIV seropositive patients: An observational study. Cureus.

[2] Ji Y, Wang Z, Shen J, Chen J, Yang J, Qi T (2017). Trends and characteristics of all-cause mortality among HIV-infected inpatients during the HAART era (2006–2015) in Shanghai, China. Biosci. Trends.

[3] Siddiqi OK, Ghebremichael M, Dang X, Atadzhanov M, Kaonga P, Khoury MN (2014). Molecular diagnosis of central nervous system opportunistic infections in HIV-infected Zambian adults. Clin. Infect. Dis..

[4] Valdez M, Moosavi L, Heidari A (2019). Concomitant central nervous system toxoplasmosis and seronegative disseminated coccidioidomycosis in a newly diagnosed acquired immune deficiency syndrome patient. J. Investig. Med. High Impact Case Rep..

[5] West CA, Chang GC, W Currie D, Bray R, Kinchen S, Behel S (2021). Unawareness of HIV infection among men aged 15–59 years in 13 Sub-Saharan African countries: Findings from the population-based HIV impact assessments, 2015–2019. J. Acquir. Immune Defic. Syndr..

[6] Sacktor N, Lyles RH, Skolasky R, Kleeberger C, Selnes OA, Miller EN (2001). HIV associated neurologic disease incidence changes: Multicenter AIDS Cohort Study, 1990–1998. Neurology.

[7] Bowen LN, Smith B, Reich D, Quezado M, Nath A (2016). HIV-associated opportunistic CNS infections: pathophysiology, diagnosis and treatment. Nat. Rev. Neurol..

[8] Thakur KT, Boubour A, Saylor D, Das M, Bearden DR, Birbeck GL (2019). Global HIV neurology: a comprehensive review. AIDS.

[9] Goyal K, Goel H, Baranwal P, Dixit A, Khan F, Jha NK (2022). Unravelling the molecular mechanism of mutagenic factors impacting human health. Environ. Sci. Pollut. Res. Int..

[10] Goel H, Goyal K, Pandey AK, Benjamin M, Khan F, Pandey P (2023). Elucidations of molecular mechanism and mechanistic effects of environmental toxicants in neurological disorders. CNS Neurol. Disord. Drug Targets.

[11] Xu XL, Zhao T, Huang YQ, Lu YQ, He XJ, Wu YS (2022). Therapeutic lumbar puncture and lumbar drainage: which is more effective for the management of intracranial hypertension in HIV patients with cryptococcal meningitis? Results of a prospective non-randomized interventional study in China. Curr. Med. Res. Opin..

[12] Wilson MR, Naccache SN, Samayoa E, Biagtan M, Bashir H, Yu G (2014). Actionable diagnosis of neuroleptospirosis by next-generation sequencing. N Engl. J. Med..

[13] Wilson MR, Sample HA, Zorn KC, Arevalo S, Yu G, Neuhaus J (2019). Clinical metagenomic sequencing for diagnosis of meningitis and encephalitis. N Engl. J. Med..

[14] Goldberg B, Sichtig H, Geyer C, Ledeboer N, Weinstock GM (2015). Making the leap from research laboratory to clinic: challenges and opportunities for next-generation sequencing in infectious disease diagnostics. MBio.

[15] Gu W, Miller S, Chiu CY (2019). Clinical metagenomic next-generation sequencing for pathogen detection. Ann. Rev. Pathol..

[16] Yang R, Zhang H, Xiong Y, Gui X, Zhang Y, Deng L (2017). Molecular diagnosis of central nervous system opportunistic infections and mortality in HIV-infected adults in Central China. AIDS Res Ther..

[17] Chen J, Zhang R, Liu L, Qi T, Wang Z, Song W (2021). Clinical usefulness of metagenomic next-generation sequencing for the diagnosis of central nervous system infection in people living with HIV. Int. J. Infect. Dis..

[18] Thakur KT (2020). Application of Pathogen Discovery/Metagenomic Sequencing in CNS HIV. Curr HIV/AIDS Rep..

[19] Shen H, Shen D, Song H, Wu X, Xu C, Su G (2021). Clinical assessment of the utility of metagenomic next-generation sequencing in pediatric patients of hematology department. Int. J. Lab. Hematol..

[20] Schlaberg R, Chiu CY, Miller S, Procop GW, Weinstock G, Professional Practice C (2017). Validation of metagenomic next-generation sequencing tests for universal pathogen detection. Arch. Pathol. Lab. Med..

[21] Wilson MR, Sample HA, Zorn KC, Arevalo S, Yu G, Neuhaus J (2019). Clinical metagenomic sequencing for diagnosis of meningitis and encephalitis. N Engl. J. Med..

[22] Luan Y, Hu H, Liu C, Chen B, Liu X, Xu Y (2021). A proof-of-concept study of an automated solution for clinical metagenomic next-generation sequencing. J. Appl. Microbiol..

[23] Basso M, Venditti C, Raponi G, Navazio AS, Alessandri F, Giombini E (2019). A case of persistent bacteraemia by *Ralstonia mannitolilytica* and *Ralstonia pickettii* in an intensive care unit. Infect. Drug Resist..

[24] Nasir N, Sayeed MA, Jamil B (2019). *Ralstonia pickettii* bacteremia: an emerging infection in a tertiary care hospital setting. Cureus.

[25] Siddiqi OK, Ghebremichael M, Dang X, Atadzhanov M, Kaonga P, Khoury MN (2014). Molecular diagnosis of central nervous system opportunistic infections in HIV infected Zambian adults. Clin. Infect. Dis..

[26] Simner PJ, Miller S, Carroll KC (2018). Understanding the promises and hurdles of metagenomic next-generation sequencing as a diagnostic tool for infectious diseases. Clin. Infect. Dis..

[27] Thakur KT (2020). Application of pathogen discovery/metagenomic sequencing in CNS HIV. Curr HIV/AIDS Rep..

[28] Bahr NC, Nuwagira E, Evans EE, Cresswell FV, Bystrom PV, Byamukama A (2018). Diagnostic accuracy of Xpert MTB/RIF Ultra for tuberculous meningitis in HIV infected adults: a prospective cohort study. Lancet Infect. Dis..

[29] Donovan J, Thu DDA, Phu NH, Dung VTM, Quang TP, Nghia HDT (2020). Xpert MTB/RIF Ultra versus Xpert MTB/RIF for the diagnosis of tuberculous meningitis: a prospective, randomised, diagnostic accuracy study. Lancet Infect. Dis..

[30] Cowan JF, Chandler AS, Kracen E, Park DR, Wallis CK, Liu E (2017). Clinical impact and cost-efectiveness of Xpert MTB/RIF testing in hospitalized patients with presumptive pulmonary tuberculosis in the United States. Clin. Infect. Dis..

[31] Xing XW, Zhang JT, Ma YB, Zheng N, Yang F, Yu SY (2019). Apparent performance of metagenomic next-generation sequencing in the diagnosis of cryptococcal meningitis: a descriptive study. J. Med. Microbiol..

[32] Gan Z, Liu J, Wang Y, Yang L, Lou Z, Xia H (2022). Performance of metagenomic next-generation sequencing for the diagnosis of cryptococcal meningitis in HIV-negative patients. Front. Cell Infect. Microbiol..

[33] Thakur KT (2020). CNS infections in HIV. Curr. Opin. Infect. Dis..

[34] Soria J, Metcalf T, Mori N, Newby RE, Montano SM, Huaroto L (2019). Mortality in hospitalized patients with tuberculous meningitis. BMC Infect. Dis..

[35] Bello-López JM, León-García G, Rojas-Bernabé A, Fernández-Sánchez V, García-Hernández O, Mancilla Rámirez J (2019). Morbidity trends and risk of tuberculosis: Mexico. Can. Respir. J..

